# miR-541-3p enhances the radiosensitivity of prostate cancer cells by inhibiting HSP27 expression and downregulating β-catenin

**DOI:** 10.1038/s41420-020-00387-8

**Published:** 2021-01-18

**Authors:** Zhenhua He, Fuhui Shen, Ping Qi, Zhenxing Zhai, Zhiping Wang

**Affiliations:** 1 Department of Neurosurgery, Lanzhou University Second Hospital, Lanzhou, 730030 China; 2 Institute of Urology, Lanzhou University Second Hospital, Lanzhou, 730030 China; 3 Department of Clinical Laboratory, Lanzhou University Second Hospital, Lanzhou, 730030 China

**Keywords:** Cell biology, Biotechnology

## Abstract

Heat shock protein 27 (HSP27), a regulator of cell survival, can enhance the resistance of cancer cells to radiotherapy. As microRNA-541-3p (miR-541-3p) was recently predicted to be a putative upstream modulator of HSP27, the present study was designed to investigate the function and mechanism underlying how miR-541-3p modulates the radiosensitivity of prostate cancer (PCa) cells by regulating HSP27. Through quantitative PCR, miR-541-3p was determined to be poorly expressed in PCa tissues relative to normal controls, whereas its expression was enhanced after radiotherapy. Consistently, miR-541-3p expression levels in PCa cells were elevated after radiation. Cell viability and proliferation and apoptosis under radiation were subsequently evaluated in response to loss-of-function of miR-541-3p. It was found that inhibition of miR-541-3p facilitated the viability and proliferation of PCa cells and promoted their apoptosis post radiation, hence reducing the radiosensitivity of LNCaP cells. Dual-luciferase reporter assay identified that miR-541-3p negatively regulated the *HSP27* mRNA expression by targeting its 3′-UTR. Meanwhile, miR-541-3p overexpression inhibited the β-catenin expression by targeting HSP27. Furthermore, HSP27 or β-catenin overexpression was noted to significantly reverse the miR-541-3p-mediated changes in the biological functions of PCa cells post radiation, suggesting that HSP27-dependent activation of β-catenin might be the mechanism responsible for the promotive effect of miR-541-3p on radiosensitivity. Collectively, this study suggests that miR-541-3p specifically inhibits the HSP27 expression and downregulates β-catenin, thereby enhancing the radiosensitivity of PCa cells. Our findings highlight the underlying mechanism of the miR-541-3p/HSP27/Wnt/β-catenin axis regarding radiotherapy for PCa.

## Introduction

Prostate cancer (PCa) is an epithelial malignant tumor that affects male populations across the world, accounting for a considerable proportion of global cancer-related deaths^1^. At present, radiotherapy plays a major curative role in the management of localized PCa, whereas it is still not possible to design a personalized radiotherapy fractionation schedule based on tumor biology due to the numerous limitations that present with the malignancy, such as radioresistance, toxicity, and relapse^2^. Currently, identification of effective biomarkers for PCa remains one of the utmost clinical challenges in the management of PCa. Despite the emergence of prostate-specific antigen (PSA) screening-powered earlier detection of PCa, the limited specificity of PSA results in its unsatisfying performance in serving as an ideal biomarker^3^. Fortunately, the hard-done work of our fellow researchers over the last decade has enriched the palette of promising biomarkers in body fluids and tissues for PCa, such as the categorization of microRNAs (miRNAs or miRs), which is endogenous noncoding RNAs capable of posttranscriptionally regulating gene expression^4,5^. Moreover, a considerable number of studies have demonstrated the dysregulation of multiple miRNAs in PCa, which suggests the use of miRNAs as potential biomarkers for the prediction and prognosis of the disease^4^. In addition, the expression of multiple miRNAs can be altered by radiation in PCa cells and surrounding cells, which are exposed in radiation treatment^6,7^. So far, several factors have been documented to be involved in the mutual effects between radiation and miRNAs: first, miRNAs could perform an inhibition role in DNA damage repair induced by radiotherapy^8^. Several miRNAs have also been reported to exhibit upregulated expressions after radiation so as to modulate the cell cycle progression^9,10^. During cell death induced by radiation, miRNAs could provoke cell cycle arrest and increase the radioresistance; furthermore, miRNAs can also regulate epithelial-mesenchymal transition (EMT)^11^, which is often affected by radiation^7,12^.

Recently, miR-541-3p, a newly identified miRNA, was reported to target the cell cycle regulator CCND1 to function as a suppressor in PCa^10^. Meanwhile, CCND1 can be regulated by various miRNAs (miR-93, miR-152-3p, and miR-16-5p) in the regulation of chemosensitivity and radiosensitivity in numerous tumors such as breast cancer, pancreatic cancer, and PCa^13–15^. Hence, it would be plausible to suggest that miR-541-3p could be another miR associated with radiosensitivity in PCa. On the other hand, heat shock protein 27 (HSP27), an important modulator for protein homeostasis and cell survival, was recently highlighted to regulate the epithelial to EMT in PCa via Wnt/β-catenin signaling modulation^16^. It has also been widely documented that HSP27 serves as an enhancer of radioresistance in human cancers such as head-and-neck and lung cancer^17,18^. Similarly, in terms of PCa progression, the Wnt signaling pathway is known to be extremely vital, especially in the development of castration-resistant PCa (CRPC), a currently incurable form of PCa^19^. In addition, Wnt/β-catenin signaling has been reported to be frequently activated in late-stage PCa, thus contributing to the development of resistance to radiotherapy^20^. As we found HSP27 as a target of miR-541-3p through bioprediction, we hypothesized that miR-541-3p could enhance radiosensitivity of PCa cells by targeting HSP27 and regulating HSP27-dependent Wnt/β-catenin. Here, to test this hypothesis, we collected PCa tissues before and after radiotherapy to determine the expression patterns of those molecules in the context of radiotherapy and identified their effects on the radiosensitivity of PCa cells. Our data finally revealed that miR-541-3p could target HSP27 and further inhibit β-catenin, thus enhancing the radiosensitivity of PCa cells. Furthermore, we carried out animal experiments and verified the possible therapeutic effect of miR-541-3p, which suggested the potential of miR-541-3p to overcome radioresistance in PCa.

## Results

### miR-541-3p, an underexpressed miR in PCa, can be enhanced after radiotherapy

**Fig. 1:**
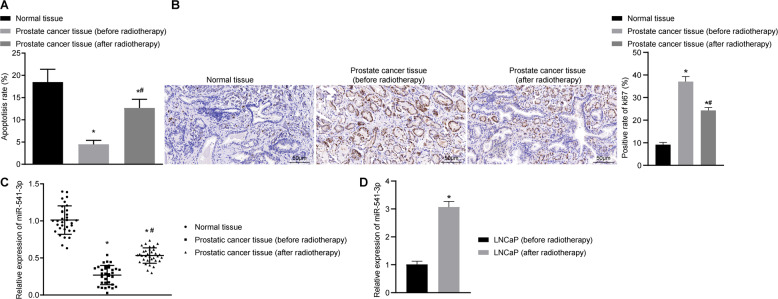
The expression of miR-541-3p is downregulated in PCa tissues but elevated after radiotherapy. **A** TACS TdT in situ apoptotic index (AI) of adjacent normal tissues and PCa tissues before and after radiotherapy. **B** The Ki67 expression in adjacent normal tissues and PCa tissues before and after radiotherapy detected by immunohistochemistry. **C** The expression of miR-541-3p in adjacent normal tissues and PCa tissues before and after radiotherapy determined by RT-qPCR. **D** The expression of miR-541-3p in LNCaP cells before or after radiation determined by RT-qPCR. Values were expressed as mean ± SEM, *p < 0.05 relative to adjacent normal tissues and #*p* < 0.05 relative to PCa tissue before radiotherapy. Paired *t*-test was used for comparison between the two groups, *n* = 33.

First, the current study investigated the expression patterns of miR-541-3p in PCa tissues and the changes in apoptosis index (AI) in PCa tissues before and after radiotherapy. A prior study has reported that miR-541-3p can delay the proliferation of PCa cells^10^. As shown in Fig. 1A, the AI value of PCa tissues before and after radiotherapy was lower compared to adjacent normal tissues (*p* < 0.05), whereas the AI value of PCa tissues after radiotherapy was higher than that before radiotherapy (*p* < 0.05). Immunohistochemistry was also performed to detect the expression patterns of proliferation marker Ki67 in adjacent normal tissues and PCa tissues before and after radiotherapy. The results (Fig. 1B) revealed that compared with adjacent normal tissues, the expression rate of Ki67 was higher in PCa tissues before and after radiotherapy (*p* < 0.05), while being reduced after radiotherapy (*p* < 0.05). Meanwhile, as reflected by reverse-transcription quantitative PCR (RT-qPCR), expression levels of miR-541-3p were significantly lower in PCa tissues before and after radiotherapy (before radiotherapy, 0.270 ± 0.130; after radiotherapy, 0.533 ± 0.104) than those in adjacent normal tissues (1.012 ± 0.193), with the lowest value detected in PCa tissues before radiotherapy (Fig. 1C). RT-qPCR results further demonstrated increased expression levels of miR-541-3p in PCa cells after radiation (Fig. 1D). These data indicated that miR-541-3p was downregulated in PCa tissues and X-ray radiation treatment promoted cell apoptosis, inhibited cell proliferation, and upregulated the expression of miR-541-3p in PCa tissues and cells.

### Overexpression of miR-541-3p increases the radiotherapy sensitivity of LNCaP cells

**Fig. 2:**
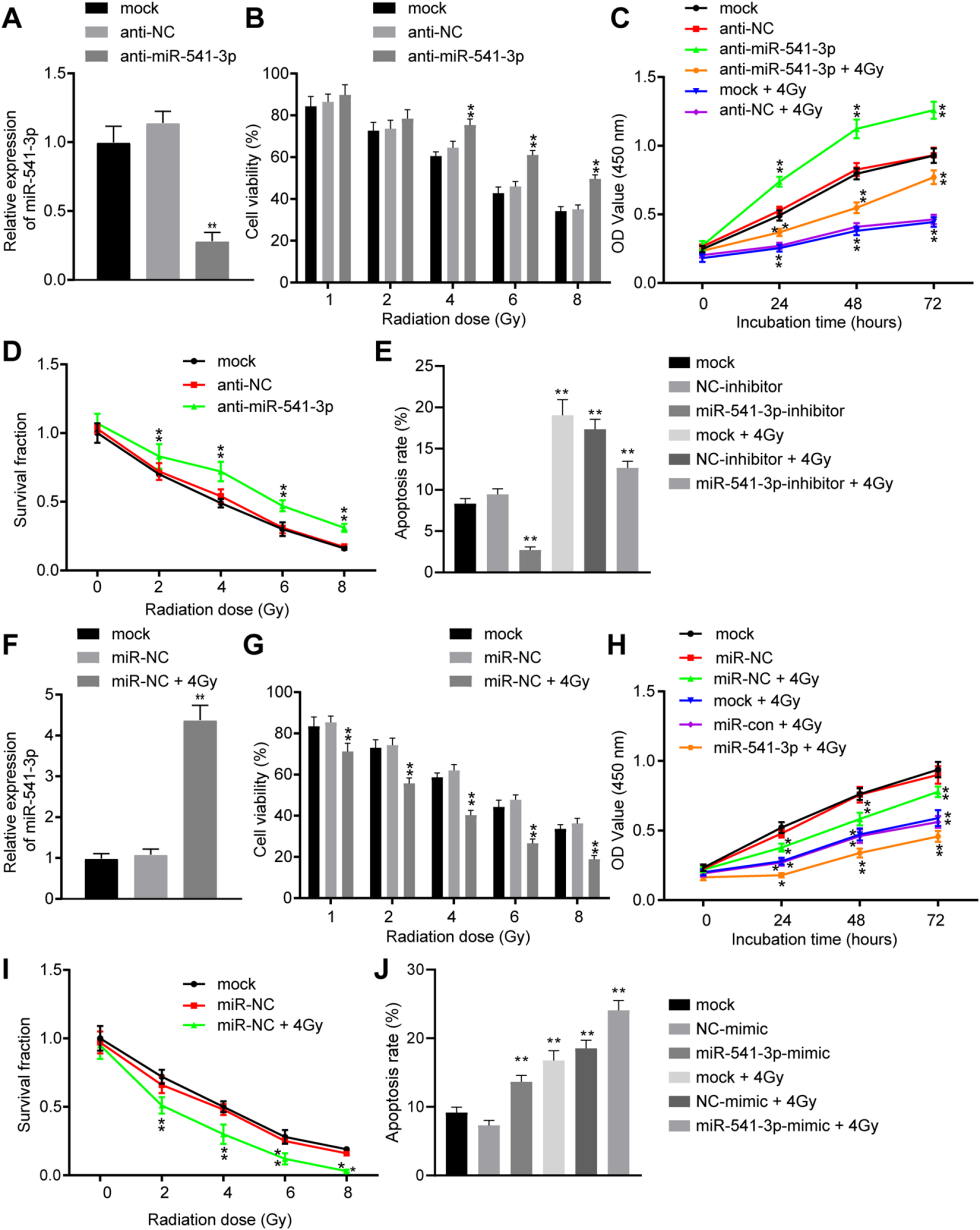
Elevation of miR-541-3p increases the radiosensitivity of LNCaP cells. **A** Expression level of miR-541-3p in LNCaP cells transfected with anti-miR-541-3p or anti-NC determined by RT-qPCR. **B** The viability of LNCaP cells after inhibition of miR-541-3p. **C** LNCaP cell proliferation after inhibition of miR-541-3p detected by CCK-8. **D** Radiosensitivity of LNCaP cells after miR-541-3p inhibition. **E** LNCaP cell apoptosis after inhibition of miR-541-3p and radiation assessed by flow cytometry. **F** Expression level of miR-541-3p in LNCaP cells after transfection with miR-541-3p mimic or miR-NC. **G** Detection of the viability of LNCaP cells after X-ray radiation and overexpressing miR-541-3p. **H** The proliferation of LNCaP cells after X-ray radiation and overexpression of miR-541-3p. **I** Colony formation ability of LNCaP cells after X-ray radiation and overexpression of miR-541-3p. **J** Cell apoptosis after X-ray radiation and overexpression of miR-541-3p. Values were mean ± expressed as SEM (*n* = 3), one-way ANOVA was used for comparison between groups, and two-way ANOVA was used for cell viability at different time points. Compared with the control group, *p < 0.05 and **p < 0.01.

After uncovering that miR-541-3p was downregulated in PCa tissues and X-ray radiation upregulated the expression of miR-541-3p in PCa, we set out to investigate the biological role of miR-541-3p in the radiosensitivity of PCa cells. At first, the expression of miR-541-3p was inhibited by means of transient transfection with anti-miR-541-3p and its negative control (NC; anti-NC) into LNCaP cells and PC3 cells (Supplementary Fig. 1). As depicted in Fig. 2A–E, inhibition of miR-541-3p attenuated the growth stagnation of radiation-induced PCa cells. Meanwhile, transient transfection with anti-miR-541-3p significantly decreased the expression levels of miR-541-3p by 71.74% in LNCaP cells (*p* < 0.01) (Fig. 2A). As shown in Fig. 2B, compared with anti-NC, inhibition of miR-541-3p promoted the cell viability under radiation of different doses, whereas viability of LNCaP cells transfected with anti-miR-541-3p began to show a significant difference under the 4 Gy radiation (*p* < 0.01). In addition, the mock-treated or transfected LNCaP cells were exposed to 0 Gy radiation (mock, anti-NC and anti-miR-541-3p) and 4 Gy radiation (mock + 4 Gy, anti-NC + 4 Gy, and anti-miR-541-3p + 4 Gy), and then cultured for 72 h. As shown in Fig. 2C, the proliferation of cells exposed to 0 Gy radiation was higher than those exposed to 4 Gy radiation. Under either 0 or 4 Gy radiation, the proliferation of cells was enhanced after transfection with anti-miR-541-3p. In addition, clonogenic assay showed that after inhibition of miR-541-3p, the colony survival of LNCaP cells significantly decreased under radiation of different doses (Fig. 2D), whereas flow cytometric results demonstrated that the apoptosis rate of cells exposed to 0 Gy was lower than those exposed to 4 Gy. Moreover, under either 0 or 4 Gy radiation, the cell apoptosis was noted to be lowered by anti-miR-541-3p (Fig. 2E). The results obtained from PC3 cells were consistent with those from LNCaP cells (Supplementary Fig. 1). These data suggested that inhibition of miR-541-3p improved the cell viability, proliferation, and colony survival, and reduced apoptosis in LNCaP cells. Further, inhibition of miR-541-3p diminished the radiation-induced growth arrest of PCa cells.

To further explore the role of miR-541-3p in response to radiation, miR-541-3p mimic was employed to enhance miR-541-3p expression in LNCaP cells. Transfection of miR-541-3p mimic was found to significantly increase the miR-541-3p levels (*p* < 0.01) (Fig. 2F). Consequently, the viability of LNCaP cells was gradually reduced under radiation in a dose-dependent manner, whereas the viability of cells was also reduced after miR-541-3p mimic transfection (Fig. 2G). In addition, the results of cell counting kit-8 (CCK-8) and flow cytometry demonstrated that miR-541-3p mimic treatment contributed to reduced cell proliferation and enhanced cell apoptosis under radiation either at 0 Gy or at 4 Gy (Fig. 2H, J). Also, the cell apoptosis under 4 Gy radiation was higher than that under 0 Gy radiation. In addition, the colony survival of LNCaP cells was noted to be gradually decreased under radiation in a dose-dependent manner, whereas the colony survival was also reduced by miR-541-3p mimic transfection under radiation (Fig. 2I). These findings suggested that overexpression of miR-541-3p reduced the cell viability, cell proliferation, and promoted apoptosis after radiation, leading to increased sensitivity of LNCaP cells to radiation.

### miR-541-3p targets and negatively regulates the HSP27 expression

**Fig. 3:**
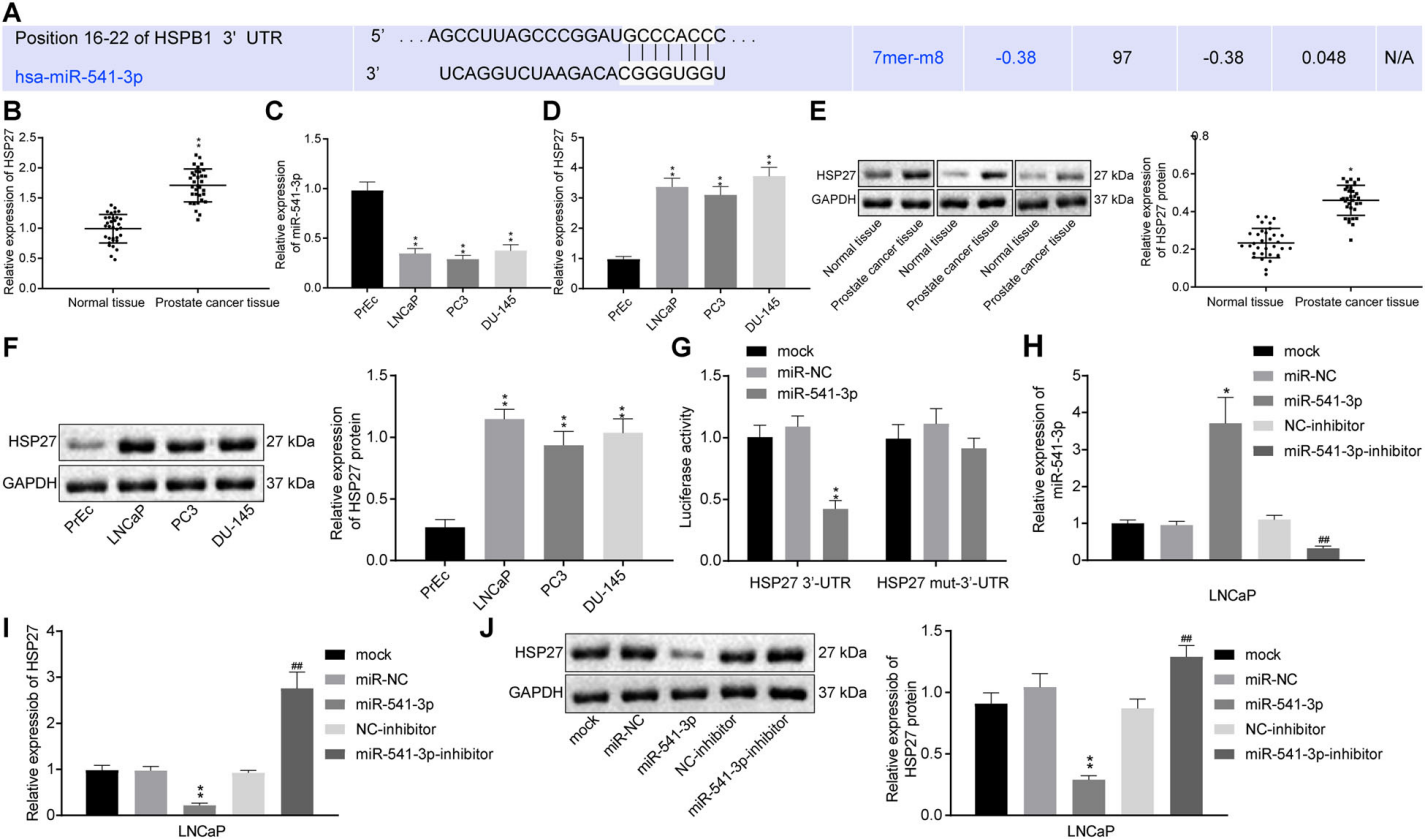
miR-541-3p targets and negatively regulates HSP27 expression. **A** Binding site of miR-541-3p and HSP27 in 3′-UTR predicted by TargetScan. **B** The mRNA expression of *HSP27* in PCa tissues and adjacent noncancerous tissues determined by RT-qPCR, **p < 0.01 relative to normal tissues (*n* = 33). **C** Expression of miR-541-3p in human PCa cell lines (LNCaP, PC3, du-145) and prostatic epithelial cells PrEc determined by RT-qPCR. **D** Expression of HSP27 in human PCa cell lines (LNCaP, PC3, du-145) and prostatic epithelial cells PrEc determined by RT-qPCR. **E** HSP27 protein expression in PCa tissues and adjacent noncancerous tissues measured by western blot assay, **p < 0.01 compared with normal tissues (*n* = 33). **F** HSP27 protein expression in human PCa cell lines (LNCaP, PC3, DU-145) and PrEc cells measured by western blot assay, **p < 0.01 relative to PrEc cells. **G** Luciferase activity in LNCaP cells 48 h after co-transfection with WT HSP27 or Mut HSP27 vector and miR-541-3p or NC. Values were expressed as mean ± SEM, compared with the control group, **p < 0.01. **H** After LNCaP cells were transfected with miR-541-3p, the expression of miR-541-3p was detected by RT-qPCR, compared with the control group, **p < 0.01. **I** After LNCaP cells were transfected with miR-541-3p, the mRNA level of HSP27 was determined by RT-qPCR, compared with the control group, **p < 0.01. **J** After LNCaP cells were transfected with miR-541-3p, HSP27 protein level were measured by western blot assay, compared with the control group, **p < 0.01. Paired *t*-test was used for comparison between the two groups and one-way analysis of variance was used for comparison between multiple groups. The values were expressed as mean ± SEM and the cell experiment was repeated for three times.

Considering the known functions of miRNAs in inhibiting the expression of their target genes^21^, we retrieved the RNAhybrid and TargetScan databases to predict the target of miR-541-3p, which identified HSP27 as a potential target of miR-541-3p (Fig. 3A).

To further determine whether HSP27 levels could be affected by the expression of miR-541-3p, the miR-541-3p expression patterns and transcriptional levels of HSP27 were first analyzed in PCa tissues, adjacent normal tissues, human PCa cell lines (LNCaP, PC3, and DU-145), and prostate epithelial cells (PrEc). RT-qPCR results showed that, compared to the adjacent noncancerous tissues, miR-541-3p expression levels were significantly lower in PCa tissues (Fig. 1C, *p* < 0.01), whereas *HSP27* mRNA levels were markedly upregulated (Fig. 3B, *p* < 0.01). Meanwhile, miR-541-3p expression levels in LNCaP, PC3, and DU-145 cells were also significantly lower than those in PrEc cells (Fig. 3C, *p* < 0.01), whereas the mRNA levels of *HSP27* were significantly higher in LNCaP, PC3, and DU-145 cells relative to PrEc cells (Fig. 3D, *p* < 0.01). Western blotting results (Fig. 3E, F) further demonstrated that the expression patterns of HSP27 protein in tissues and cell lines, which were consistent with the results of RT-qPCR.

In addition, to clarify whether miR-541-3p was involved in the regulation of HSP27, LNCaP cells were co-transfected with miR-541-3p mimic and pGL3-HSP27, the 3′-untranslated region (3′-UTR) or pGL3-HSP27 mutants (MUT), and dual-luciferase reporter assay was used to determine whether miR-541-3p could target the HSP27 plasmids. The results showed that the luciferase signal in cells co-transfected with miR-541-3p mimic and pGL3-HSP27 3′-UTR was markedly decreased (Fig. 3G, *p* < 0.01), whereas that in cells transfected with pGL3-HSP27 mut-3′-UTR exhibited no marked differences. These results indicated that miR-541-3p could bind to the 3′-UTR region of HSP27, thereby inhibiting the transcription and expression of HSP27.

The transcriptional expression levels of miR-541-3p and HSP27 in LNCaP cells were further determined using RT-qPCR and western blot assay after miR-541-3p mimic or anti-miR-541-3p transfection. The results showed that miR-541-3p levels were significantly upregulated, whereas HSP27 mRNA and protein levels were downregulated in response to miR-541-3p mimic transfection in LNCaP cells (*p* < 0.01); however, HSP27 mRNA and protein levels could be elevated by miR-541-3p inhibition (Fig. 3H–J). These results suggested that miR-541-3p could target and inhibit the expression of HSP27.

### miR-541-3p enhances the radiosensitivity of PCa cells by inhibiting HSP27 and further downregulating β-catenin

**Fig. 4:**
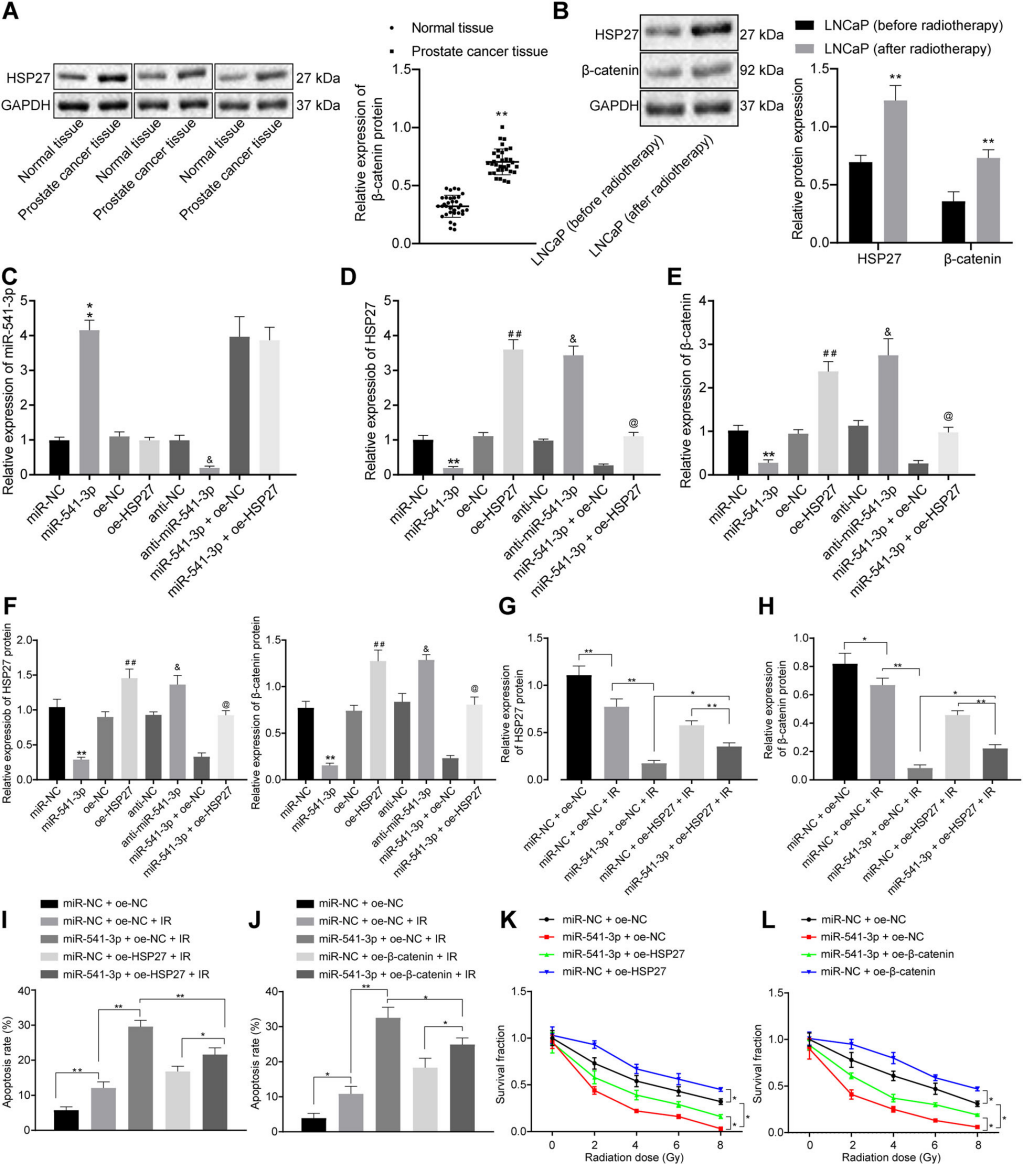
miR-541-3p enhances the radiosensitivity of PCa cells through downregulating β-catenin by inhibiting HSP27. In **C**–**F**, miR-NC, miR-541-3p, oe-NC, and oe-HSP27 were transfected into LNCaP cells. **G**–**J** The effect of HSP27 and β-catenin on the radiosensitivity mediated by miR-541-3p in LNCaP cells. **A** The protein expression of β-catenin in PCa tissues and adjacent normal tissues measured by western blot assay. **p < 0.01 relative to the adjacent normal tissues. **B** The protein expression of HSP27 and β-catenin in LNCaP cells with or without radiation measured by western blot assay. **p < 0.05 relative to LNCaP cells after 0 Gy radiation. **C** The expression level of miR-541-3p determined by RT-qPCR. **D** The mRNA expression of *HSP27* determined by RT-qPCR. **E** The expression level of *β-catenin* mRNA was detected by RT-qPCR. **F** The protein expression levels of HSP27 and β-catenin measured by western blot assay, **p < 0.01 relative to the miR-NC and ^##^*p* < 0.01 relative to the oe-NC. ^&^*p* < 0.01 relative to anti-NC; ^@^*p* < 0.01 relative to miR-541-3p + oe-NC. **G** HSP27 protein expression level measured by western blot assay, **p < 0.01, compared with the miR-NC, ^##^*p* < 0.01. **H** The protein expression level of β-catenin measured by western blot assay, **p < 0.01, compared with the miR-NC, ^##^*p* < 0.01. **I** The effects of HSP27 overexpression on miR-541-3p-mediated apoptosis rate of LNCaP cells assessed by flow cytometry. **J** The effects of β-catenin overexpression on miR-541-3p-mediated apoptosis rate of LNCaP cells assessed by flow cytometry. **K** Effects of overexpression of HSP27 on the miR-541-3p-mediated radiotherapy sensitivity of LNCaP cells assessed by clonogenic assay. **L** Effects of overexpression of β-catenin on the miR-541-3p-mediated radiotherapy sensitivity of LNCaP cells assessed by clonogenic assay. One-way ANOVA was used for the comparison between groups and two-way ANOVA was used for the analysis of cell viability at different time points. The values were expressed as mean ± SEM (*n* = 3).

Numerous studies have shown that upregulation of β-catenin promotes the tolerance to radiotherapy in PCa^22^, whereas HSP27 is known to inhibit the binding between GSK3 and β-catenin, and thus prevent the degradation of β-catenin by proteasomes^16^. Therefore, we investigated whether miR-541-3p could affect the protein stability of β-catenin by regulating the expression of HSP27 in LNCaP cells. Initially, protein expression patterns of β-catenin were found to be markedly increased in PCa tissues compared with adjacent normal tissues (Fig. 4A). In addition, we observed significantly higher protein expression levels of HSP27 and β-catenin in the LNCaP cells exposed to 4 Gy radiation compared with those exposed to 0 Gy radiation (Fig. 4B, *p* < 0.01).

LNCaP cells were further transfected with miR-NC, miR-541-3p mimic, oe-NC, oe-HSP27, and miR-541-3p mimic + oe-HSP27, and the expression patterns of miR-541-3p, HSP27, and β-catenin in LNCaP cells were detected using RT-qPCR and western blot analyses. As shown in Fig. 4C–F, overexpression of miR-541-3p led to significant downregulation of HSP27 and β-catenin mRNA and protein levels in LNCaP cells, whereas miR-541-3p inhibition resulted in elevations in these molecular levels. Conversely, compared with the oe-NC transfection, HSP27 and β-catenin mRNA and protein levels were significantly elevated in response to oe-HSP27 transfection (Fig. 4D-F). Meanwhile, overexpression of HSP27 also rescued the expression of β-catenin inhibited by miR-541-3p in LNCaP cells. These results suggested that miR-541-3p inhibited the expression of β-catenin by downregulating HSP27.

Further experiments were conducted to investigate the effects of HSP27 and β-catenin on the radiosensitivity mediated by miR-541-3p in LNCaP cells. As shown in Fig. 4G, H, radiation at 4 Gy was found to induce the downregulation of HSP27 and β-catenin (*p* < 0.01). Interestingly, when miR-541-3p was overexpressed, a larger downregulation was detected in either HSP27 or β-catenin expression levels. However, compared with the radiation-exposed LNCaP cells transfected with miR-541-3p mimic, the expression of HSP27 and β-catenin proteins was significantly upregulated in the radiation-exposed LNCaP cells co-transfected with miR-541-3p mimic + oe-HSP27 (*p* < 0.05), whereas β-catenin protein expression was also upregulated in the radiation-exposed LNCaP cells co-transfected with +miR-541-3p mimic + oe-β-catenin (*p* < 0.05). Results in Fig. 4I, J showed that under radiation at 4 Gy, overexpression of HSP27 or β-catenin weakened the miR-541-3p-mediated enhancement in apoptosis of LNCaP cells. In addition, clonogenic assay results (Fig. 4K, L) showed that overexpression of HSP27 or β-catenin attenuated the miR-541-3p-mediated inhibition of colony survival of LNCaP cells, indicating that downregulation of HSP27 and β-catenin was responsible for miR-541-3p-mediated enhancement of radiosensitivity.

### miR-541-3p enhances the radiosensitivity of PCa in mouse xenograft models

**Fig. 5:**
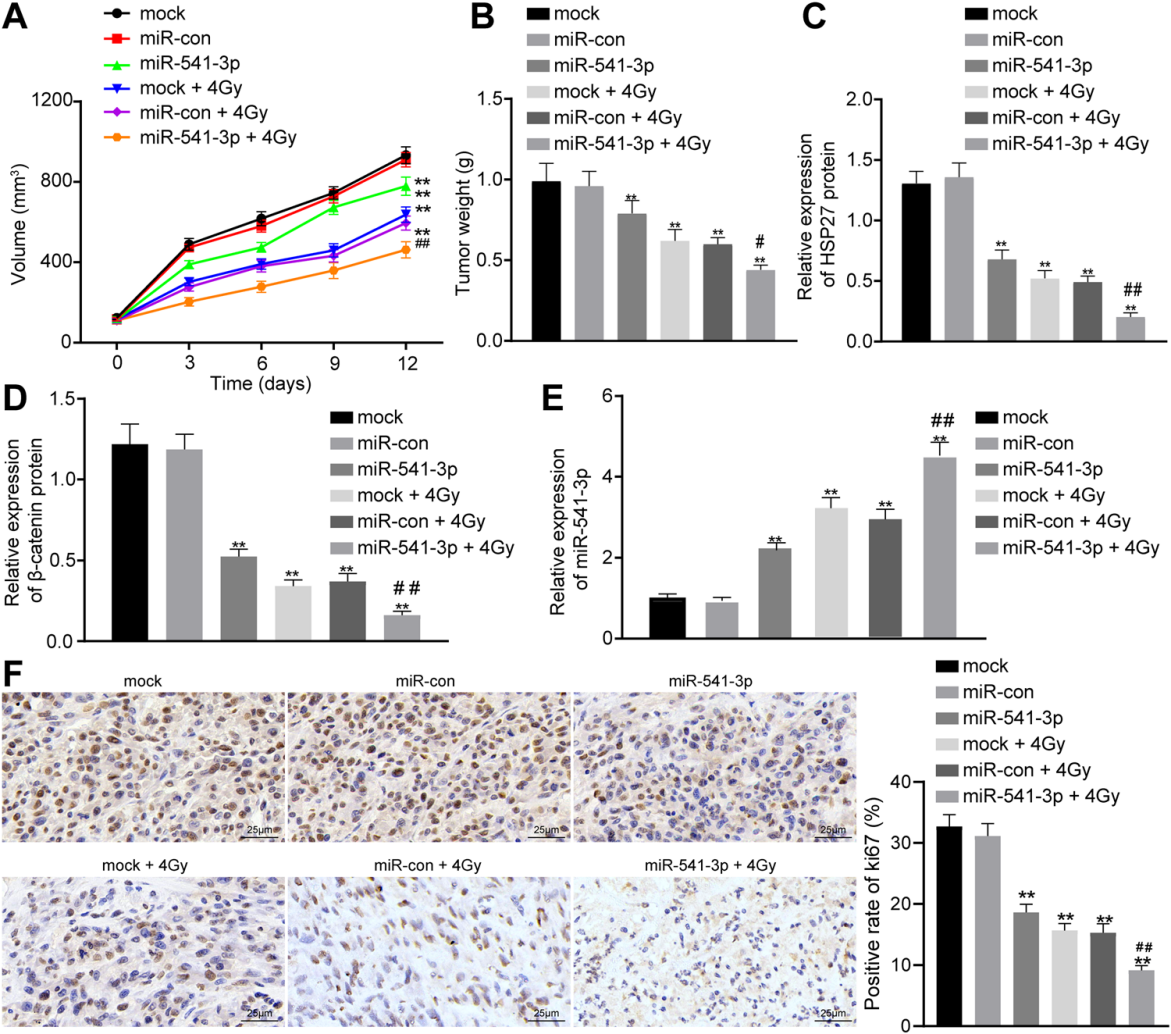
miR-541-3p enhances radiosensitivity of PCa cells in xenograft tumor model. LNCaP cells with and without miR-NC and miR-541-3p transfection were subcutaneously injected into the nude mice. Fourteen days after injection, the xenograft sites were exposed to a single dose of 4 Gy X-ray radiation using Faxition 43885D X-ray machine. The tumor size was measured every 2 days using a caliper. The xenograft tumor was dissected 12 days after radiation, weighed, and lysed for western blot assay. **A** Tumor size measured with caliper. **B** The weight of xenograft tumors. **C** HSP27 protein expression in tumor tissues measured by western blot assay. **D** The expression level of β-catenin protein in tumor tissues measured by western blot assay. **E** The expression level of miR-541-3p in tumor tissues determined by RT-qPCR. **F** The Ki67 expression rate in tumor tissues of nude mice detected by immunohistochemistry. **p < 0.01 relative to mock + 0 Gy radiation, ^#^*p* < 0.05 relative to mock + 4 Gy radiation, and ^##^*p* < 0.01 relative the mock + 4 Gy radiation; one-way analysis of variance was used for comparison between groups and repeated-measurement analysis of variance was used for tumor volume at different time points, with the values represented as mean ± SEM (*n* = 5).

Lastly, to determine the radiosensitivity mediated by miR-541-3p to PCa in vivo, we performed animal experiments. As shown in Fig. 5A, 12 days after 4 Gy X-ray exposure, tumors in the mice injected with mock-treated LNCaP cells or miR-NC-treated LNCaP cells continued to grow. However, tumor growth in the nude mice was found to be significantly attenuated by enhancement of miR-541-3p expression in vivo. In addition, the results in Fig. 5B showed that compared with the tumor weight under 0 Gy radiation, the tumor weight after 4 Gy radiation was generally reduced and could be significantly lowered by enhancement of miR-541-3p expression. Compared with 0 Gy radiation, 4 Gy radiation significantly inhibited the expression levels of HSP27 and β-catenin protein but increased those of miR-541-3p (Fig. 5C–E). Under 4 Gy radiation, additional enhancement of miR-541-3p in nude mice markedly reduced the expressions of HSP27 and β-catenin (*p* < 0.01).

Immunohistochemistry was further performed to measure the Ki67 expression rate in the nude mice to evaluate cell proliferation. Results revealed that 4 Gy radiation reduced the expression rate of Ki67, whereas elevation of miR-541-3p also downregulated the Ki67 expression in tumor tissues (Fig. 5F).

Altogether, these findings indicated that miR-541-3p appreciably delayed tumor growth after radiotherapy, which was consistent with the radiosensitizing effect of miR-541-3p in vitro.

## Discussion

Radiotherapy is regarded as the treatment of choice for patients suffering from localized PCa^23^. However, the emergence of radioresistance and local recurrences remain the most significant clinical problems, ultimately restricting the clinical outcomes of PCa treatments^24^. In the current study, we uncovered that miR-541-3p exhibited increased expression levels in PCa tissues when exposed to X-ray radiation. In addition, elevating the expression of miR-541-3p brought about a suppressive effect on the viability and proliferation of LNCaP cells after radiation, thus augmenting the radiosensitivity. Further analysis revealed that miR-541-3p could target the HSP27 mRNA *via* its 3′-UTR to inhibit the expression of β-catenin. Lastly, we also validated the above findings by means of nude mice experimentation, wherein miR-541-3p could significantly inhibit the tumor growth after radiation.

First, findings obtained in our study demonstrated that miR-541-3p was poorly expressed in PCa tissues. Similarly, downregulated expression of miR-541-3p has been previously documented in other malignancies such as hepatocellular carcinoma (HCC)^25^ and non-small cell lung cancer (NSCLC)^26^. In addition, we observed that miR-541-3p could enhance the radiosensitivity in PCa, therefore, our results on miR-541-3p enriches the palette of miRNAs in enhancing radiosensitivity for PCa and also provides a new potential biomarker for the radiotherapy of PCa. Meanwhile, our findings also indicated that the expression levels of HSP27 and β-catenin were upregulated in PCa tissues. β-Catenin is known as a key effector of Wnt signaling, which is critical for the regulation of tissue homeostasis, whereas the aberrant activation of Wnt/β-catenin has been documented in multiple cancers, including colorectal cancer^27^, oral squamous cancer^28^, as well as PCa^19,29^. As for PCa, several miRNAs such as miR-744 and miR-182, are capable of promoting tumor progression via abnormal activation of Wnt/β-catenin signaling^30,31^. Moreover, reports also suggest that Wnt/β-catenin signaling activation facilitates ductal morphogenesis in fetal prostate^32^. In terms of HSP27, its overexpression was previously associated with poor outcomes and EMT in PCa, which is very much in accordance with our findings^33,34^. HSP27 has also been identified as a key effector of the progression of many other cancers. For instance, the upregulation of HSP27 has been reportedly to be associated with breast cancer^35^, and other tumor types^36^. Especially in PCa, HSP27 was highlighted to participate in epidermal growth factor (EGF)-mediated EMT via modulation of the β-catenin-related signaling pathway, wherein HSP27 could enhance the stability of β-catenin, thus enabling a mutual regulatory role between HSP27 and β-catenin in PCa^16^. Together, these findings and evidence indicate that miR-541-3p could enhance the radiosensitivity in PCa and the high expression levels of HSP27 and β-catenin during the course of disease.

Further analysis in the current study demonstrated that miR-541-3p could target HSP27 3′-UTR. Similarly, Long et al.^10^ illustrated that miR-541-3p can target the cell cycle regulator CCND1 in PCa, through which miR-541-3p could regulate the proliferation and cell cycle progression of PCa cells, whereas our findings revealed an unrecognized target for miR-541-3p in PCa. Multiple studies have also associated the levels of HSP27 with the progression of PCa by virtue of EMT promotion^16,34^. To date, most PCa-related deaths are attributed to the progression of PCa to the lethal metastatic disease upon the development of CRPC^37^, whereas EMT is known to endow malignant cells with increased migratory and survival attributes to facilitate tumor progression. More notably, HSP27 has been highlighted to play vital roles in promoting metastasis of PCa *via* modulating EGF-mediated EMT. When EGF activates epidermal growth factor receptor (EGFR), the downstream signaling pathways such as PI3K/AKT and MAPK/ERK can be stimulated, which further confer important roles in metastasis and tumor progression^38^. After that, AKT can induce the phosphorylation of β-catenin and promote the transcription of β-catenin-related genes^39^, whereas some of these genes also possess the ability to induce EMT in cancers^40^. In addition to tumor progression, inhibition of HSP27 was previously demonstrated to enhance radiosensitivity in head-and-neck cancer through regulating DNA repair and lung cancer by modulating the stress response protein Redd1^17,18^. In the current study, we found that overexpression of miR-541-3p could inhibit the expressions of HSP27 and β-catenin, thus enhancing the radiosensitivity of PCa. As a result, our discoveries provide an unrecognized piece of evidence that miRNAs could enhance radiosensitivity through Wnt-related pathways, thus enriching the modulation mechanisms of miRNAs in PCa.

Moreover, we further validated the capability of enhancing radiosensitivity by miR-541-3p in PCa by means of in vivo experimentation, which was manifested by suppressed tumor growth after radiation treatment in nude mice. Despite previous researches demonstrating that miR-541-3p can play tumor-inhibitory roles in HCC, NSCLC, and PCa, none of them carried out in vivo experiments to confirm the suppressive effects of miR-541-3p^25,26^. Herein, by using xenograft mice, we provided the first piece of evidence that highlights the ability of miR-541-3p to maintain the regulatory role at animal levels, which also encourages a further step for the applications of these findings to the radiotherapy of PCa. Under the context revealed by this study, future applications based on miR-541-3p could be developed to monitor the treatment efficacy of the radiotherapy.

**Fig. 6:**
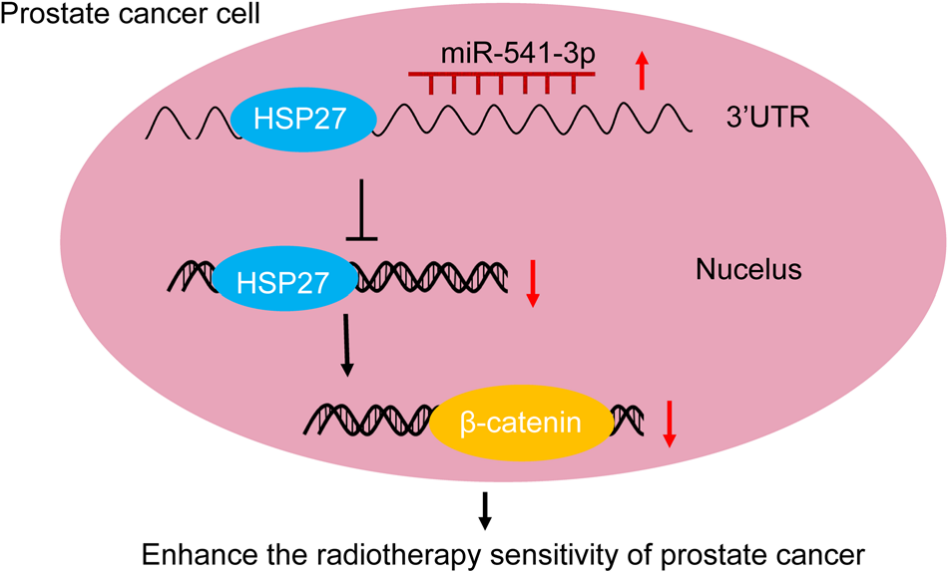
The molecular mechanism diagrams. miR-541-3p-mediated inhibition of HSP27 enhances the radiosensitivity of PCa cells by downregulating the expression of β-catenin.

In summary, the current study identified that miR-541-3p was capable of enhancing the radiosensitivity of LNCaP cells (Fig. 6). In addition, a new target of miR-541-3p, HSP27, was also identified by means of dual-luciferase reporter assay, which could further modulate β-catenin and thus perform the radiosensitization effect. Likewise, the results obtained from xenograft mouse models were consistent with the radiosensitization effects identified in LNCaP cells. Therefore, our findings suggest that miR-541-3p may contribute to the therapeutic benefit to treating PCa by overcoming the existent radioresistance. Collectively, our results provided theoretical basis to support the further clinical investigation in developing novel therapeutic approaches based on the miR-541-3p/HSP27/β-catenin signaling pathway to combat radioresistance in PCa.

## Materials and methods

### Clinical samples

PCa tissues and adjacent normal tissues (at a distance from tumor tissues ≥ 5 cm) were collected from 33 patients via radical resection at the Lanzhou University Second Hospital, with an age range of 45–70 years. Signed informed consents were obtained from all patients prior to sample collection. All experimental procedures were approved by the Ethics Committee of the Lanzhou University Second Hospital and at the same time, complied with the Declaration of Helsinki. None of the included patients underwent chemotherapy or radiotherapy before surgery. Tumor tissues before radiotherapy and adjacent normal tissue samples were obtained during surgical resection and quickly frozen in liquid nitrogen. Also, tumor tissues after radiotherapy were collected after treatment and stored at −80 °C for further experimentation.

### Cell culture and tissue collection

Human PCa cell lines LNCaP, DU-145, PC3, PrEC, and human embryonic kidney cells (HEK293A) were procured from Shanghai Cell Bank of China Academia Sinica, Shanghai, China. The obtained cells were cultured in Roswell Park Memorial Institute-1640 (Gibco, Grand Island, NY, USA) with 10% fetal bovine serum, and penicillin and streptomycin (100 U/mL) for cell culture at a temperature of 37 °C and 5% CO_2_.

### Radiation

X-ray radiation was produced at 100 kVp using an X-ray machine (Faxitron RX-650, USA). The cells were irradiated at a dosage of 0.835 Gy/min at ambient temperature with unirradiated cells set as controls.

### In situ apoptosis assay

TACS TdT in situ apoptosis detection kits (R& D Systems) were utilized to determine the apoptosis rate in the tissues. Briefly, the tissue slices were de-paraffined and rinsed with phosphate buffer saline (PBS). The sections were then digested with protease K at 24 °C for 15 min. After washing, the endogenous peroxidase activity was quenched using methanol solution with 3% H_2_O_2._ The slides were subsequently incubated with TdT-labeled reaction mixture at 37 °C for 1 h. The color was developed using the TACS (blue)-labeled substrate solution. Finally, the slides were washed, re-stained and fixed with Permount solution.

### Flow cytometric analysis of apoptosis

The cells were seeded in a 96-well plate, at a density of 2 × 10^4^/mL. Each well was plated with 100 µL cell suspension, and the plate was cultured in a humidified incubator for 24 h. Upon attachment, the cells were irradiated with 4 Gy of X-ray radiation. Subsequently, the apoptosis rates were detected using fluorescein isothiocyanate Annexin V apoptosis detection kits (BD Pharmingen, San Jose, CA, USA) after 48 h of radiation.

### Cell transfection

Cells at the logarithmic phase of growth were trypsinized and then seeded in a six-well plate, at a density of 1 × 10^5^ cells per well. After routine culture for 24 h, the cells were transfected with miR-541-3p mimic, miR-NC, anti-miR-541-3p, and anti-NC (all synthesized by GenePhama, Shanghai, China) according to the instructions of Lipofectamine 2000 kits (Invitrogen, USA), and GV214 (GeneChem Co., Ltd, Shanghai, China). The full-length sequence of HSP27 or β-catenin was amplified by PCR and subcloned into the pcDNA3.1 vector (Invitrogen, USA) to generate oe-HSP27 or oe-β-catenin plasmid. The medium was replaced 6 h after transfection.

### Cell viability assessment

First, 1 × 10^3^ cells were seeded in 96-well plates with 6 parallel wells per group. After 12 h, the cells were treated with 4 Gy X-ray. After 48 h, 10 µL 3-(4,5-dimethylthiazol-2-yl)-2,5-diphenyl tetrazolium bromide (5 mg/mL) (Sigma, USA) was added to each well and incubated at 37 °C for 4 h. Post-incubation, the supernatant was removed and 150 ml dimethyl sulfoxide (Sigma) was added to each well. Lastly, the absorbance value (optical density) of each well was measured at a wavelength of 490 nm.

### Cell proliferation analysis

Cells were seeded in 24-well plates, at a density of 5 × 10^3^ cells per well, with 4 parallel wells per group. Next, 10 µL CCK-8 was added to the medium and the cells were incubated at 37 °C for 4 h. Subsequently, the absorbance was measured at a wavelength of 450 nm.

### Clonogenic assay

Cells at the logarithmic phase of growth were rinsed twice with PBS and resuspended. After cell counting, the cells were diluted and seeded in a plate with a diameter of 60 mm according to different radiation doses (triplicates for each dose). Next, the cells were irradiated with 0, 2, 4, and 6 Gy, and incubated for 10–14 days. During incubation, the medium was replaced one to two times. The cells were then rinsed with PBS, fixed with methanol for 20 min, and stained with 0.1% crystal violet for 15 min. After the medium was dried, the number of colonies with more than 50 cells was counted under a low power microscope. The plating efficiency (PE) and survival fraction (SF) were calculated according to the following formula: PE = (number of colonies in the blank group/number of cells seeded in the blank group) × 100%, SF = number of colonies/(number of the seeded cells × PE).

### Luciferase assay

The TargetScan database (http://www.targetscan.org/) was retrieved to search for potential targets of miR-541-3p, which indicated HSP27 to be a target of miR-541-3p. To further investigate whether miR-541-3p directly targeted HSP27, we utilized dual-luciferase reporter assay. The 3′-UTR of HSP27 was amplified using PCR, and the 3′-UTR mutation product of HSP27 (mut-3′-UTR) was synthesized by site-directed mutation. After cleavage with restriction enzymes, the amplified PCR product was cloned into the polyclonal site of the reconstructed pGL3 expression vector. The pRL-TK control vector expressing *Renilla* luciferase (Promega, Madison, WI, USA) was utilized for normalization of transfection efficiency. Finally, the luciferase activity was detected using Dual-Luciferase® Reporter assay kits (Promega, Madison, WI, USA).

### Western blot assay

The tissues and cells were lysed with a pH 7.4 radio-immunoprecipitation assay lysis buffer (1% Triton X-100, 10 mM Tris, 1 mM EDTA, 1 mM EGTA, 150 mM NaCl) supplemented with protease inhibitors (1:100) and centrifuged at 12,000 r.p.m. at 4 °C. Next, the protein concentration was measured with a BioRad Bradford protein assay kit and followed by sodium dodecyl sulfate-polyacrylamide gel electrophoresis. The proteins were subsequently transferred onto a polyvinylethylene fluoride membrane and incubated continuously for 1 h in TBST containing 5% bovine serum albumin (25 mmol/L Tris pH 7.5, 150 mmol/L NaCl, and 0.1% Tween 20). Afterwards, the membranes were incubated with antibodies to HSP27 (catalog number: 2442S, dilution ratio of 1:1000), β-catenin (catalog number: 9587T, dilution ratio of 1:1000), and GAPDH (catalog number: 5174S, dilution ratio of 1:1000) from Cell Signaling Technology (Beverly, MA, USA) overnight at 4 °C and then reacted with a horseradish peroxidase-labeled secondary antibody (Santa Cruz Biotechnology, Santa Cruz, CA, USA) for 1 h. After each incubation, the membrane was thoroughly washed with TBST three times. Lastly, the blots were developed with an enhanced chemiluminescence detection reagent.

### Reverse-transcription quantitative PCR

First, total RNA content was extracted from the samples using miRNeasy Mini kits (Qiagen, Valencia, CA, USA) and cDNA was synthesized with the help of TaqMan MicroRNA Reverse Transcription kits (Applied Biosystems, Foster City, CA, USA). Real-time qPCR was performed using TaqMan MicroRNA analysis kits (Applied Biosystems). For HSP27 and β-catenin, total RNA extraction and real-time qPCR were performed using the SYBR GreenER™ two-step kit (Invitrogen, Carlsbad, CA, USA). All the aforementioned operations are performed in accordance with the manufacturer’s instructions. Real-time PCR was performed using a 7500 RT-PCR instrument (Applied Biosystems, USA). PCR was conducted according to the following procedures: 50 °C for 2 min, 95 °C for 10 min, 95 °C for 50 cycles of 15 s, 60 °C for 1 min. The primer sequences are listed in Table 1. Each experiment was repeated three times.

**Table 1:** Primer sequence.

Gene	Sequence
miR-541-3p	F 5′-GTGTAACCACATCCTCGACTGA-3′
	R 5′-GATTAGTGCCGTGGAGAAG-3′
*U6*	F 5′-GCTTCGGCAGCACATATACTAA-3′
	R 5′-AACGCTTCACGAATTTGCGT-3′
*HSP27*	F 5′-ACGAAGAAAGGCAGGATGAA-3′
	R 5′-GATGGGTAGCAAGCTGAAGG-3′
*β-catenin*	F 5′-AAG TTC TTG GCT ATTACG ACA-3′
	F 5′-ACA GCA CCT TCA GCA CTCT-3′
*GAPDH*	F 5′-GAAGGTGAAGGTCGGAGTC-3′
	R 5′-GAAGATGGTGATGGGATTTC-3′

### Xenograft tumor in nude mice

Initially, 6-week-old BALB/c male nude mice were purchased from Chongqing Tengxin Biotech., Co., Ltd, Chongqing, China. The tumor mice were maintained in a laboratory animal center under specific-pathogen-free conditions. All animal experiments were conducted in accordance with the guidelines for the care and use of experimental animals, and all experimental programs were approved by the Animal Ethics Committee of Lanzhou University Second Hospital. To establish PCa xenografts in nude mice, LNCaP cells, GV214-miR-con, or GV214-miR-531-3p-transfected LNCaP cells were suspended in a 1:1 mixture of matrix gel (BD Biosciences) and medium, and injected subcutaneously into the mice under sterile conditions (randomly grouped in a blinded manner, five mice per group). After 14 days of injection, the tumor site was exposed to a single dose of 4 Gy X-ray radiation using the Faxition 43885D X-ray machine. The tumor size was measured every two days with Vernier calipers within 12 days after radiotherapy, and the tumor volume was calculated according to the following formula: (long axis × short axis 2)/2. The xenograft tumor was removed 12 days after radiotherapy and weighed after dissection.

### Immunohistochemistry

The specimens were fixed with 10% formaldehyde, paraffin-embedded, and sliced into 4 μm sections. Next, the sections were placed in a 60 °C incubator for 1 h, dewaxed with xylene, and dehydrated with gradient ethanol. Afterwards, the sections were incubated in 3% H_2_O_2_ (Sigma) at 37 °C for 30 min, and then placed in 0.01 M citric acid buffer. Following boiling at 95 °C for 20 min, the sections were sealed with normal goat serum at 37 °C for 10 min. The sections were reacted with rabbit anti-Ki67 (ab16667, dilution ratio of 1:1000; Abcam, Cambridge, UK) at 4 °C for 12 h, and then reacted with the corresponding biotin-labeled goat anti-rabbit secondary antibody at ambient temperature for 10 min, followed by incubation with horseradish peroxidase-labeled streptavidin for 10 min at and color development with diaminobenzidine. Later, the sections were stored in a dark room for 8 min, stained with hematoxylin, dehydrated, cleared, mounted, and observed under a light microscope. Finally, the positive cells were counted using the Nikon image analysis software in 3 non-repetitive fields of equal area.

### Statistical analysis

Statistical analyses were performed using the SPSS 21.0 software (IBM, USA). Measurement data were summarized by mean ± SD. Paired *t*-test was used for comparison of experimental data among PCa tissues before and after radiotherapy and adjacent normal tissues, and unpaired *t*-test was performed for comparisons between other two groups. One-way analysis of variance and Tukey’s post-hoc test were used for comparisons among multiple groups. Two-way analysis of variance and Bonferroni correction were used to test the cell viability at different time points. A value of *p* < 0.05 was regarded statistically significant.

## Supplementary information

Supplementary Figure 1
