# Supplementary figures and images for: miR-541-3p enhances the radiosensitivity of prostate cancer cells by inhibiting HSP27 expression and downregulating β-catenin
Source: Cell Death Discov. 2021 Jan 18;7:18. doi: 10.1038/s41420-020-00387-8 (PMC7813831; doi:10.1038/s41420-020-00387-8)

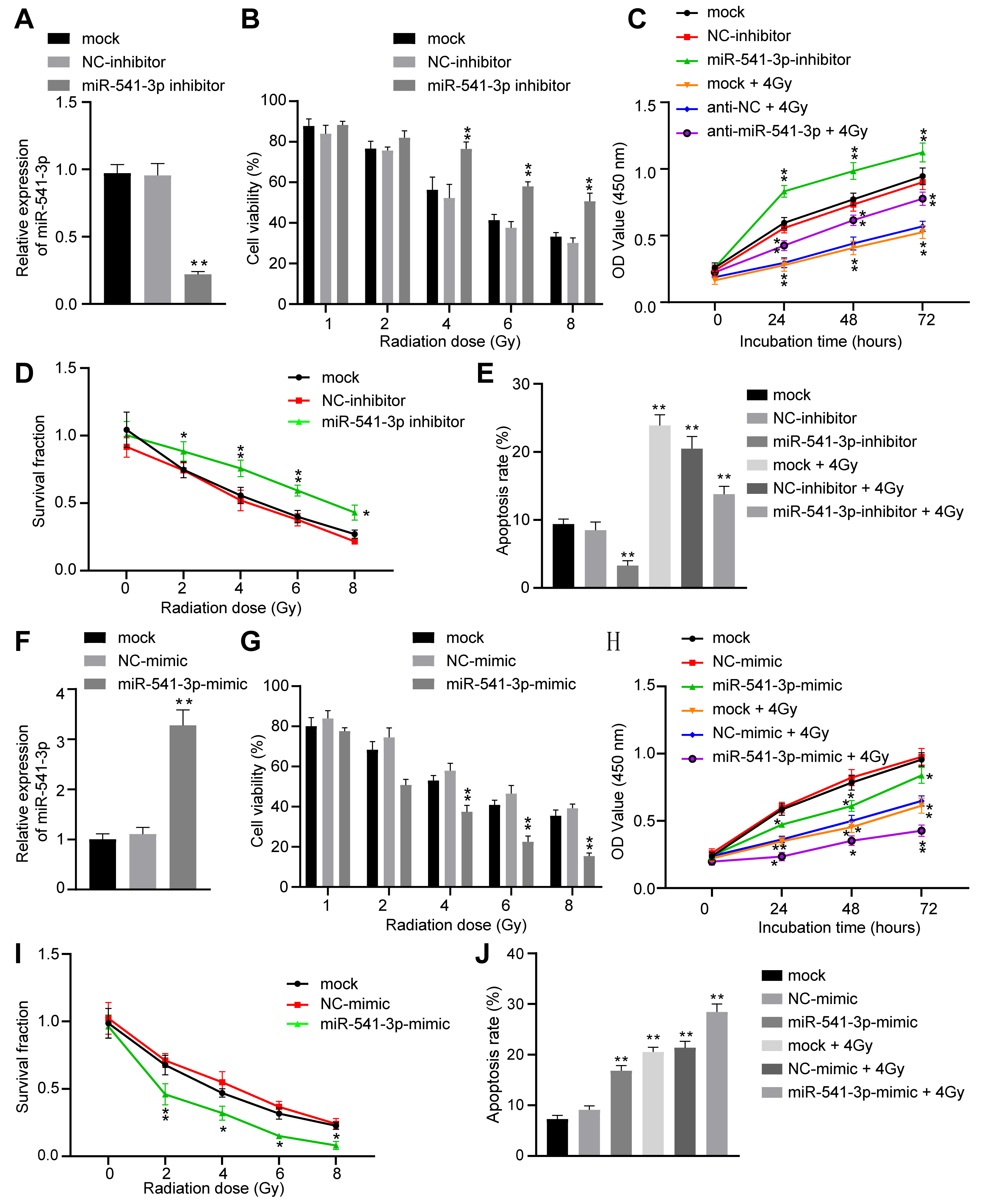

Supplement: Supplementary file 1 — Supplementary Figure 1 [file 41420_2020_387_MOESM1_ESM.tif]
